# Something must happen before first breath

**DOI:** 10.1186/s12910-021-00624-4

**Published:** 2021-05-12

**Authors:** Daniela Polese, Marcella Fagioli, Fabio Virgili, Paolo Fiori Nastro

**Affiliations:** 1 Department of Mental Health and Sensory Organs (NESMOS), Sant’Andrea University Hospital, Sapienza University of Rome, Rome, Italy; 2 ASL Rome 1, Rome, Italy; 3 Department of Sciences, University Tor Vergata, Rome, Italy; 4 School of Medicine and Dentistry, Sapienza University of Rome, Rome, Italy

**Keywords:** First breath, Human birth, Foetus, Brain’s development, Newborn, Infant, Brain’s activation, Extrauterine environment

## Abstract

**Background:**

Definition and concept of the ‘beginning of human life’ are weakened by co-existing contrasting hypotheses based on humanistic or religious beliefs rather than scientific foundations. This plethora of conceptually distant views have important common concerns in different fields of science and shape, in turn, several societal aspects including laws related, for instance, to inheritance eligibility or abortion, end-of-life care and euthanasia, and reproductive technology. Also, they are fundamental to evaluate opportunity for resuscitation vs. palliative care in extremely preterm infants. In this article, we address one of the most common tenets in medicine: the acceptance that human life starts with first breath, even though several events are well-documented to take place before its occurrence.

**Main text:**

Several studies show how pivotal physiological events take place before first breath. Evidence of a number of neurological events occurring before first breath opens the way to the primacy of the Central Nervous System, given its immediate extra-uterine activation at birth. This activation eventually sets specific physiological conditions that allow the complex sequence of events determining the muscle activity associated with the influx of air in the lung and the settling of a continuous and successful extra-uterine respiration. We would like to invite the scientific community to endorse a clear-cut position against the paradigm of ‘first breath’ as the beginning of life. Herein, we also assume how, a still undefined, yet possibly specific *quid* in the external environment triggers further physiological response in newborns. Better understanding of the critical events that occur at the beginning of human life is likely to cause great concern and expectations in scientists, researchers and physicians working in the domain of brain, and its physiology, and mental health.

**Conclusions:**

The comparison between beliefs and evidence-based observations generates confusion, misperceptions and false expectations in society, hence, in the scientific and medical community. Different and more solid alternatives about the carachterization of the ‘beginning of human life’ are indeed available and require to be explored and defined.

## Background

Given all of the technical and ethical limitations, human birth remains a complex topic difficult to be investigated. The beginning of human life^1^ is set by the transition from intrauterine condition to extrauterine environment and corresponds with the status of being born [1]. Lack of in-depht knowledge about the dynamics of human birth causes common concerns in different fields of science, as, on the one hand, they drive basic medical decisions and, on the other, affects society and its rules and regulations, from family issues to abortion, from end-of-life care to euthanasia, and reproductive technology. As an illustrative example regarding the optimization of decisional protocols in neonatal clinical practice, a definite agreement on this theme is evidently pivotal to provide a robust sharable evaluation of the opportunity to resuscitate, rather than only provide palliative care, at the limits of viability, i.e. extremely preterm newborns [2]. Beyond this evident specific concern, lack of shared consensus within the scientific community and society on the very beginning of human life is evident and requires to be solved.

Although several different neonatologists admit that they have never thought that the beginning of human life is linked to first breath, this tenet remains very popular. This debate is aimed at overcoming this specific principle and intends to raise the point about the need for a shared, science-based agreement on the beginning of human life. The flaw is not novel. For instance, more than 60 years ago, on *The Lancet*, the medical community interrogated itself about this theme, concluding that, “The first breath of a new-born infant marks the climax of a dramatic physiological upheaval, but the precise sequence of events is still far from clear” [3]. Nowadays, it seems that no significant progresses in any direction have been made. Herein, we put forth some evidence on what occurs, before first breath takes place, in terms of maturation of specific anatomical structures and the occurrence of other physiological events, which are not currently evaluated or measured, but are indicative of infants’ viability.

## Main text

### A definition of the beginning of human life

The concept that the breakthrough between foetus and new-born is determined by the occurrence of a first respiratory action affects different medical fields, such as paediatrics, obstetrics and neonatology. While brain inactivity is widely accepted and acknowledged as the operative condition to decide life-end, definition of the initial extreme of human life still relies on the common, popular idea that life begins with ‘first breath’, despite the fact that several different events have been shown to take place beforehand. The mechanisms that are at the basis of the beginning of human life are expected to be multifactorial and still not well-characterized. As a consequence, the regions/structures of the brain that are needed for regular brain functioning at birth have still not been clearly or definitively identified.

WHO Guidelines consider that, rather than immediately at birth, first breath can physiologically occur up to 60 s after birth[4]. This delay varies inter-individually, averaging to about 20 s and has been well-documented by several reports [5, 6].

This observation entails two different alternatives: either the newborn is not actually ‘alive’ in the first few seconds of extrauterine existence or human life begins with something else, an unidentified *quid*, a trigger that is responsible for the initial stimulation of the complex cascade that precedes and leads to first breath.

A recent paper by André and co-workers [7] addresses the newborn’s subjective perception of a totally new environment at birth, which is defined as the *Umwelt*^2^ by carefully and accurately identifying changes in the external surrounding. André and co-workers also highlight the existence of a significant discontinuity between the intrauterine and post-natal sensorial activities, which strongly suggests that the Central Nervous System (CNS) plays a pivotal role in the passage from one condition to the other, generating specific physiological responses that characterize human birth and ‘the beginning of human life’. Starting from what has been observed [7], we suggest that this *quid* is at the basis of an extremely rapid activation of the CNS following sensorial stimulation by the new environment. This activation of the CNS, in turn, triggers the neuro-muscular activity that is needed for first breath.

### Physiological events that lead to first breath: the primacy of the Central Nervous System at birth

First respiratory action must necessarily be preceded by the clearance of the fluid from the lungs. The adrenaline-mediated activation of sodium channels on the apical pulmonary surface stops the secretion of the fluid and initiates its reabsorption [8]. Clearance process happens simultaneously with labour, with uterine contractions that causes foetal chest wall to change, thereby driving the fluid outside the lungs. Approaching term, the release of pulmonary surfactant by alveolar cells lowers air-to-liquid interfacial tension, thereby facilitating the expansion of the lungs. All these steps start before first respiratory action and make it possible. Also, in order to take place, first breath needs active contraction of the diaphragm, which causes an expansion of thoracic cage, a dilation of intra-thoracic trachea and the movement of air into the lungs. Contraction is controlled by multiple factors and stimuli, all originating in diverse areas of the CNS, and involves many afferent and efferent neural arcs, including volitional, sensory, and biochemical inputs as well as motor outputs to respiratory muscles, facial structures, and airway effectors. All these signals are integrated, modulated and transmitted to effector organs by the brain [9]. This explains why brain death is associated with the abrogation of autonomous breathing function. Recent clinical studies have shown that, at birth, abrupt activation of the Kölliker-Fuse pontine nucleus takes place and initiates the first inspiratory action [10]. This happens via direct stimulation of the facial/parafacial complex that, in turn, activates the pre-Bötzinger nucleus, which is the actual activator of the diaphragm. At birth, motoneurons allow for the generation of more substantial movements of the thoracic cage that can thus be expanded to meet new breathing requirements *ex utero* [11].

Upstream of the above-mentioned well-known physiological events, the very first trigger of the activation of the brain has still not been identified nor characterized. Capability to react to sensory stimuli is key to viability of both pre-term and term newborns, as also measured by the Apgar score [12, 13]. Only when neural connections between sense organs and the cortex have been established, which corresponds with the formation of thalamo-cortical connections (23–24th GW) [14], does the foetus become potentially able to react to sensory stimuli and therefore viable. Moreover, based on Virginia Apgar’s method, the presence of heart rate at birth is considered as a sign of life and demands manoeuvres for resuscitation. However, after resuscitation, the presence of the capability to react to stimuli, with higher Apgar scores, establishes that the infant is improving and is likely to survive.

Significant discontinuity between the intrauterine and postnatal environment and complete novelty of the latter [7] suggests how the CNS, which is sensitive to the external stimulation, plays a pivotal role in the passage from one stage to the other, characterising human birth and ‘the beginning of human life’. This rapid transition opens the way to the possibility that actual trigger for fully-developed brain activation may be immediate stimuli from the *Umwelt.*

Evidence of a number of neurological events occurring before first breath opens the way to the primacy of the CNS at birth, due to an ‘extrauterine’ activation induced by a necessary but still undefined specific *quid* in the environment. This activation is expected to set the basis for the occurrence of all those specific physiological conditions that lead to events determining the influx of air in the lungs, first breath and continuous and successful extra-uterine breathing. We strongly believe that knowledge about the physiological events that occur at birth, once they have been acknowledged by the scientific community and accepted by society, would help both physicians and parents to decide whether and when to resuscitate extremely preterm infants [2].

## Conclusions

The popular tenets of human life starting with first breath as the landmark of human birth can be considered a matter of ‘practical consensus’. However, we strongly believe that, given its relevance for basic medical decisions, this question should rely on something more robust than that. Just for the sake of the argument, one could claim that death occurs when an individual stops breathing, which is utterly false. This point of view, however, brings us back to the above-mentioned controversy: when does ‘life’ start? A presence of a plethora of distant views about this topic [1] spoils and jeopardize several aspects of everyday practical medicine and of everyday life, as mentioned above. Unlike evidence-based observations, traditional tenets risk to generate confusion, misperceptions and false expectations in both the scientific community and society.

Unfortunately, scientific knowledge is replaced by mythical substitutes, such as religious and popular beliefs, generating a vicious circle that needs to be broken.

This debate does not want to offer immediate solutions or generate immediate shared consensus. Rather, it is aimed at promoting discussions between peers, from neurobiology to neonatologists, psychologists and psychiatrists, about the importance of the brain’s reaction to external stimuli. To this end, we invite readers with different interests and expertise, and different degree of familiarity with the scientific literature we make reference to, to comment the pertinence and relevance of papers called to support our hypothesis, according to their specific background.

There is great need for studies about this initial *quid,* based on which new protocols on viability and resuscitation of preterm newborns could be defined. For instance, advancements in relevant research are expected to provide instruments that can help identify specific structures’ functions and/or activation (i.e. Kölliker-Fuse pontine nucleus, facial/parafacial complex, thalamo-cortical connections) and measurable parameters to establish viability and, thus, drive medical actions. In this perspective, we consider that medical consensus should rely on a solid scientific platform. Decisions, as is often the case in critical situations, cannot just be rule of thumb.

Indeed, consensus on the central role of the CNS activation at birth would reconcile science with the definition of ‘end of life’, which is associated with brain inactivity rather than loss of autonomous breathing or of the heart function, as it has been established by the Harvard Medical School’s protocol [15].

The hypothesis of a necessary, immediate reactivity of the newborn brain to the new environment, preceding and leading any subsequent physiological response, sounds more than plausible. In particular, the extrauterine surrounding, with its new and strong stimuli, is likely to trigger the reaction of the CNS, when the newborn is suddenly exposed to it. Further research is needed in this field to better understand the mechanisms that are at the basis of cerebral activity and how the brain relates to the external environment throughout human beings’ life.

In conclusion, we believe that the scientific community should endorse a clear-cut position against the paradigm of the ‘first breath’ as the beginning of life. Better knowledge about several critical events occurring at birth is likely to cause great concern but also great expectation in scientists, researchers and physicians working in the domain of brain, and its physiology, and mental health [16]. We hope that our notes will encourage our colleagues to contribute to this discussion. A ‘call for consensus’ is definitively needed.

## Data Availability

All data generated or analysed during this study are included in this published article.

## Abbreviations

CNS: Central Nervous System
GW: Gestational weeks
WHO: World Health Organization

## Footnotes

1 For the sake of this discussion, we set as baseline the existence of a *caesura *between intrauterine and extrauterine condition, and the term ‘life’ will only be assigned to the latter. This assumption excludes a priori any possible ambiguity relevant to the concept of ‘life before birth’ and, at the same time, the idea of a ‘spiritual’ origin of life, which is scientifically unacceptable.

2 “individuals’ subjective universe determined by their sensory perceptions and cognitive abilities”
